# 217. Eosinophil count recovery as a predictor of bloodstream clearance in *Staphylococcus aureus* bacteremia

**DOI:** 10.1093/ofid/ofad500.290

**Published:** 2023-11-27

**Authors:** Jack W McHugh, Ryan B Khodadadi, Zach Yetmar, Andrew Halvorsen, Omar M Abu Saleh

**Affiliations:** Mayo Clinic, Rochester, Minnesota; Mayo Clinic, Rochester, Minnesota; Mayo Clinic, Rochester, Minnesota; Mayo Clinic, Rochester, Minnesota; Mayo Clinic Rochester, Rochester, Minnesota

## Abstract

**Background:**

Eosinopenia has previously been studied as a predictor of bacteremia and sepsis, and has been identified as a marker of increased mortality in critically ill patients. To date, one study has examined changes in eosinophil count during bacterial infection, noting quicker normalization of eosinophil count versus traditional markers of inflammation, whereas no studies have examined eosinophil count recovery as a predictor of blood stream clearance.

**Methods:**

We conducted a multicenter, retrospective cohort study of adult patients diagnosed with *Staphylococcus aureus* bacteremia (SAB) between January 2018 and December 2019 at Mayo Clinic campuses. Patients with eosinopenia on the date of the first positive blood culture were selected for further analysis. Eosinopenia was defined as an eosinophil count < 0.03 x10(9)/L. Eosinophil count recovery was defined as the first subsequent date where an eosinophil count ≥0.03 x10(9)/L was recorded. Clearance of SAB was defined as negative blood cultures at 48 hours on two consecutive days, and the date of clearance was defined as the date of first negative blood culture.

**Results:**

A total of 213 cases of SAB were identified where an eosinophil count was obtained on date of first positive blood culture. Of these cases, 132 (62.0%) had eosinopenia at time of diagnosis. Data related to count recovery was available for 90 patients, with associated characteristics for this cohort detailed in Table 1. *S. aureus* isolates were oxacillin susceptible in 91.1% (82/90) of cases. The median time to clearance of bacteremia was 2.7 days (IQR 1.8-4.7 days), and the median time to eosinophil count recovery was 2.0 days (IQR 2.0-3.0 days). Data related to timing of eosinophil count recovery are presented in Table 2. Resolution of eosinopenia occurred within 48 hours of bloodstream clearance in 79/90 (87.8%) of cases and preceded or occurred within 48 hours of blood culture clearance in 88/90 (97.8%) of cases.

**Figure:**
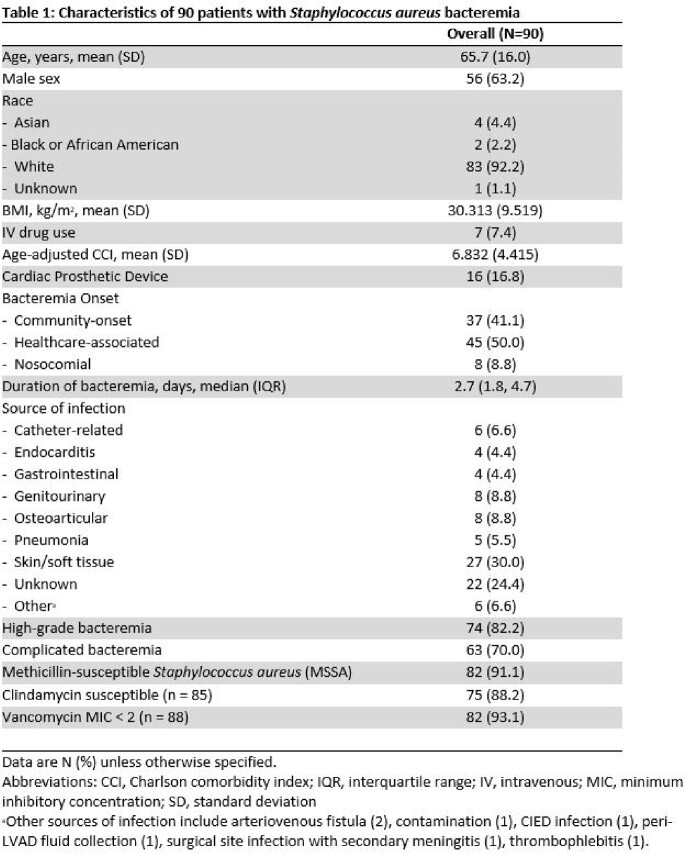

**Figure:**
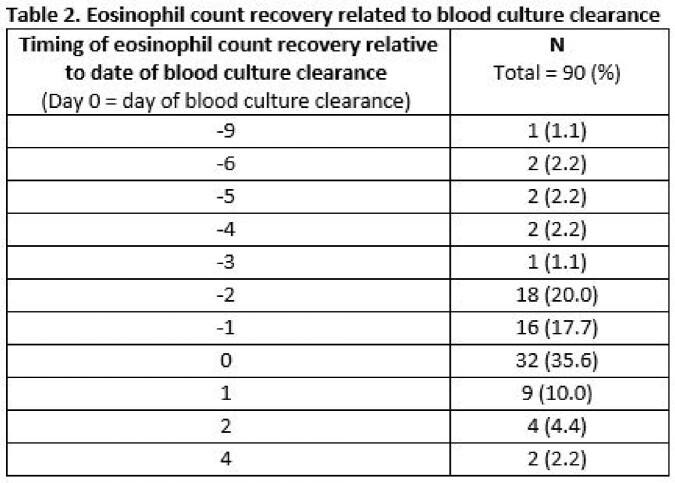

**Conclusion:**

Eosinopenia is a common finding at time of diagnosis of SAB, and recovery of eosinophil count is suggestive of imminent clearance of blood cultures.

**Disclosures:**

**All Authors**: No reported disclosures

